# The Human Gut Chip “HuGChip”, an Explorative Phylogenetic Microarray for Determining Gut Microbiome Diversity at Family Level

**DOI:** 10.1371/journal.pone.0062544

**Published:** 2013-05-17

**Authors:** William Tottey, Jeremie Denonfoux, Faouzi Jaziri, Nicolas Parisot, Mohiedine Missaoui, David Hill, Guillaume Borrel, Eric Peyretaillade, Monique Alric, Hugh M. B. Harris, Ian B. Jeffery, Marcus J. Claesson, Paul W. O'Toole, Pierre Peyret, Jean-François Brugère

**Affiliations:** 1 EA CIDAM 4678, Clermont-Université, Université d'Auvergne, Clermont-Ferrand, France; 2 CNRS, UMR 6158, ISIMA/LIMOS, Aubière/Clermont-Ferrand, France; 3 Department of Microbiology and Alimentary Pharmabiotic Centre, University College Cork, Cork, Ireland; Auburn University, United States of America

## Abstract

Evaluating the composition of the human gut microbiota greatly facilitates studies on its role in human pathophysiology, and is heavily reliant on culture-independent molecular methods. A microarray designated the Human Gut Chip (HuGChip) was developed to analyze and compare human gut microbiota samples. The PhylArray software was used to design specific and sensitive probes. The DNA chip was composed of 4,441 probes (2,442 specific and 1,919 explorative probes) targeting 66 bacterial families. A mock community composed of 16S rRNA gene sequences from intestinal species was used to define the threshold criteria to be used to analyze complex samples. This was then experimentally verified with three human faecal samples and results were compared (i) with pyrosequencing of the V4 hypervariable region of the 16S rRNA gene, (ii) metagenomic data, and (iii) qPCR analysis of three phyla. When compared at both the phylum and the family level, high Pearson's correlation coefficients were obtained between data from all methods. The HuGChip development and validation showed that it is not only able to assess the known human gut microbiota but could also detect unknown species with the explorative probes to reveal the large number of bacterial sequences not yet described in the human gut microbiota, overcoming the main inconvenience encountered when developing microarrays.

## Introduction

The human gut harbours a complex ecosystem composed of 10^14^ microbial cells [1], including eukaryotic and archaeal cells [2], [3]. Although a high inter-individual diversity is present and is modulated by several factors [4]–[6], a phylogenetic core at the species level was hypothesized [7]:composed of 66 Operational Taxonomic Units (OTUs) which were present in more than 50% of the individuals and which represented about 36% of the total sequences. More than 1,500 different bacterial species have already been associated with the human gut microbiota and around 500 different bacterial species constitute an individual human gut microbiota [8]. Furthermore, it has been shown that the gut microbiota impacts upon the health of its host, for example by influencing the maturation of the immune system, by modulating the barrier function the gut epithelium and by conferring colonization resistance or direct antagonism protection against pathogens [9]. It also provides a set of metabolic functions which are not present in the coding capacity of human organism, such as the digestion of some resistant carbohydrates, energy storage or the production of vitamins [10]. Furthermore, the gut microbiota has also been reported to play a major role in diseases like colon cancer [11], obesity [12], inflammatory bowel disease [13], [14] or cardiovascular disease [15]. Over the last two decades, development of culture independent techniques has significantly increased our knowledge of gut microbiota. Tools permitting exhaustive analysis of individual gut microbiota including a phylogenetic identification and (semi-) quantification are still under development. Most of these techniques are based on the 16S ribosomal RNA (rRNA) gene sequence variations between different species. Fluorescence In Situ Hybridization (FISH) and fingerprinting techniques such as Denaturing Gradient Gel Electrophoresis (DGGE), Terminal Fragment Length Polymorphism (T-RFLP) are frequently used (reviewed in [16]). However, they generally lack resolution and do not allow high-throughput direct phylogenetic identification. More recently techniques such as DNA microarray hybridization and next-generation sequencing (NGS) have been developed granting further phylogenetic identification of microbiota diversity [16], [17].

Microarray technology is a high throughput platform used to study numerous samples and to detect thousands of nucleic acids sequences simultaneously making it fast and user friendly. Phylogenetic DNA microarrays consist of several thousand probes, usually designed from rRNA gene sequence database targeting either specific organisms (e.g. pathogenic bacteria) or the whole microbiota at various taxonomic levels. The use of 16S rRNA microarrays provides superior diagnostic power compared to clone library techniques [18]. Several microarrays addressing the gut microbiota have been developed over the last decade, showing differences in their design and the aims of study. In 2007, Palmer and colleagues designed an array containing 10,265 probes, each spotted once, and targeting 1,629 species [19]. Another microarray addressing the whole gut microbiota was published by Paliy *et al.* (2009) and was spotted with 16,223 probes targeting 775 bacterial species [20]. Finally, the Human Intestinal Tract Chip (HITChip) was designed to target 1,140 species using 4,809 overlapping probes [21]. More recently, array hybridization results were compared to pyrosequencing of the V1 to V6 hypervariable regions of the 16S rRNA gene sequence and showed a good correlation [22], [23]. The authors suggested that the differences observed between the data from the two techniques might arise from a combination of the analysis of different hypervariable regions, the limited number of 16S rRNA gene sequences available for the probe design, and the ability of these probes to only target known 16S rRNA gene sequences.

Phylogenetic microarray probe design can be performed using various software packages such as ARB [24], PRIMROSE [25] and ORMA [26] which have been widely used as they provide specific and sensitive probes to address sequences from databases. In spite of the exponential growth of data within international databases, our current understanding of microbial diversity is still incomplete. Microarrays coupled with explorative probe design strategies are, therefore, well suited to survey complete microbial communities, including microorganisms with uncharacterized sequences [27]. The PhylArray [28] and the KASpOD [29] probe design software were developed to provide sensitive, specific and also explorative probes dedicated to phylogenetic microarrays [28]. This innovative probe design strategy may help to overcome the main limitation of microarrays i.e. the inability to detect unknown sequences and thus, to survey uncharacterized microbial populations.

In this study, we present the Human Gut Chip (abbreviated in HuGChip), a novel phylogenetic microarray. It is designed using the PhylArray software, and is intended to assess the human gut microbiota at the family level using 4,441 25-mer probes representing 66 families present in the human gut microbiota.

## Materials and Methods

### Ethics statement

This study was approved by the Clinical Research Ethics Committee of the Cork Teaching Hospitals: Informed written consent was obtained from all ELDERMET subjects or, in cases of cognitive impairment, by next-of-kin in accordance with the local research ethics committee guidelines, the Clinical Research Ethics Committee of the Cork Teaching Hospitals.

### Human faecal samples, bacterial strains and nucleic acids extractions

Total DNA was extracted from three human faecal samples using Qiagen's DNA Stool Kit (Qiagen, West Sussex, UK) and adjusted to 10 ng/µl. All DNA quantifications were performed using NanoDrop ND-1000 spectrophotometer (NanoDrop Technologies, Wilmington, DE). In order to prepare a mock community (16S rRNA bacterial amplicons), the bacterial strains *Lactobacillus acidophilus* (ATCC 4356), *Escherichia coli* (S123), *Clostridium coccoides* (ATCC 29236), *Clostridium leptum* (ATCC 29065) and *Bacteroides fragilis* (DSM 2151^T^) were used. Total genomic DNA was extracted from pure bacterial cultures using DNeasy Blood and Tissue Kit (Qiagen, West Sussex, UK) and concentration was adjusted to 10 ng/µl to be used as 16S rRNA gene PCR amplification templates.

### Microarray probe design and production

**Figure 1:**
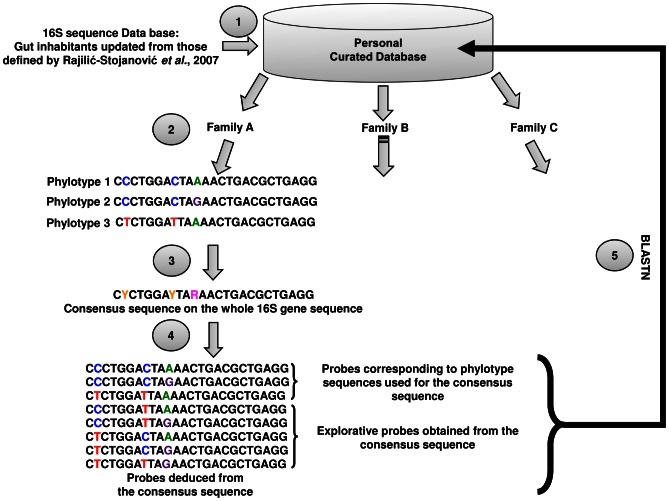
Probe design procedure using the PhylArray software (adapted from [**27**] **).** (1) The creation of a database was an essential part of the procedure; making sure this database contained good quality, correctly affiliated sequences was crucial. (2) The selection of a targeted taxonomic level and the reorganisation of the sequences so that they belonged to the correct taxon. (3) For each different taxon (e.g. family), a consensus sequence on the whole 16S gene sequence was constituted with all the sequences it contained. (4) The software then tested all the possible probe regions on the whole sequence using a 25 nucleotide sliding window with a step of 1 nucleotide. It selected the 5 regions with the best specificity and degeneracy for each taxon and developed all the probe combinations. (5) Finally, the software verified probe specificity performing a nucleotide BLAST against the initial database which allowed to distinct the specific from the explorative probes.

The DNA microarray was designed using a custom 16S rRNA gene database. This was derived from the sequences described in 2007 by Rajilić-Stojanović *et al.* [8] and consisted of 1,052 sequences (longer than 1,000 nucleotides) which can be accessed at http://g2im.u-clermont1.fr/HuGChip/. The PhylArray software was used to design 25-mer probes [28]. The first step of the PhylArray algorithm (Figure 1) is the extraction of all available sequences corresponding to the targeted family from our custom 16S rRNA curated database. Retrieved sequences are then aligned using the ClustalW program [30]. A degenerate consensus sequences is then deduced from this multiple alignment, taking into account the sequence variability at each position. Degenerate candidate probes are then selected along the consensus sequence, and all non-degenerate combinations are checked for cross-hybridizations against the 16S rRNA database. The locus corresponding to each 25-mer degenerate probe is referred to hereafter as a “region”. Among the combinations derived from each degenerate probe, some correspond to sequences that have not yet been deposited in the databases, namely explorative probes. Such probes should, therefore, allow the detection in this environment of undescribed microorganisms belonging to the targeted taxon. The best 5 “regions” of each consensus sequence, harbouring the best specificity for the taxon were selected to represent the taxon. Finally, these selected probes were subsequently verified by BLASTN [31] against the two other databases (Greengenes [32], SILVA [33]) containing microbial sequences from many different kinds of ecosystems. The microarray was synthesized by Agilent Technologies (Agilent Technologies, Palo Alto, CA) using the *in situ* surface attached synthesis [34] with a multiplex format of 8×15k where each probe was randomly spotted in three replicates across the array to reduce biases caused by spatial variations.

### 16S rRNA gene PCR amplification

16S rRNA genes were amplified using universal primers 27F (AGAGTTTGATCMTGGCTCAG) and 1492R (TACGGYTACCTTGTTACGACT) [35]. PCR reactions were performed in a 50 µl volume, in the presence of 10 ng of template DNA, using DreamTaq DNA polymerase (Fermentas, St. Leon-Rot, Germany). The PCR reaction consisted of an initial denaturation step at 95°C for 5 min followed by 35 cycles of denaturation at 95°C for 30 s, annealing at 58°C for 40 s and elongation at 72°C for 2 min. A final extension step was performed at 72°C for 5 min. PCR product size was verified by electrophoresis with 1% (w/v) agarose gel and were purified using the MinElute PCR Purification Kit (Qiagen Ltd., UK) following manufacturer's instructions and stored at −20°C. The purified amplicons from the bacterial strains were then mixed to a final amount of 1 µg of DNA composed of 100 ng of *L. acidophilus* and *E. coli*; 200 ng of *C. coccoides*; 250 ng of *B. fragilis* and 350 ng of *C. leptum* forming the mock community.

### Sample labelling and microarray hybridization, reading and analysis

For each sample (faecal samples and the mock community), the non-fragmented purified 16S rRNA gene PCR products (1 µg) were labelled with either Cy3 or Cy5 using the Genomic DNA ULS labelling Kit (Agilent Technologies, Palo Alto, CA) following the manufacturer's instructions. For microarray hybridization, 100 ng of labelled artificial bacterial DNA mix and 250 ng of each labelled faecal sample were used (GEO accession number GSE44752). Hybridization was performed following the Agilent OligoaCGH hybridization protocol (Agilent Technologies, Palo Alto, CA) at 65°C for 24 h. Microarray washings were performed as recommended by Agilent and slides were scanned at a 3-µm resolution using a Surescan microarray scanner (Agilent Technologies, Palo Alto, CA). Pixel intensities were extracted using the “Feature Extraction” software (Agilent Technologies, Palo Alto, CA). The retained intensity value for each probe was the spot's median intensity signal. For each probe, the median value of its replicates was calculated and was further identified as the “probe signal”. For each of the 5 regions (considering every bacterial family), the highest probe signal was selected as the more representative probe and characterized the “region signal”. For each family, a mean signal of the five “region signals” was calculated providing the “family signal”. It was then used to determine the relative abundance of each family by dividing it with the sum of all the “family signals”. Specific scripts developed in this study with the Delphi and the C++ languages were used to automatically perform these data extractions.

### V4 16S rRNA gene pyrosequencing and metagenomic analyses of the samples

DNA extracted from three human faecal samples from the ELDERMET project (samples 176, 204 and 205) was analyzed by 454 pyrosequencing of the 16S rRNA V4 region amplicons on a 454 Genome Sequencer FLX Titanium platform as described by Claesson *et al.* [22]. Two of these samples (176 and 205) were also analysed by direct random shotgun sequencing of libraries with 91 bp paired-end Illumina reads and 350 bp insert size, further assembled using MetaVelvet [36] as described by Claesson *et al.* [5]. Raw metagenomic data are available at the MG-RAST server [37] with the following reference number 4491484.3 and 4491423.3. To determine the microbiota composition from the metagenomic samples, the rRNA sequences were affiliated using the RDP, SILVA and Greengenes database with a maximum E-Value cut-off of 1e^−5^, a minimum percentage identity cut-off of 80% and a minimum alignment length cut-off of 50 nucleotides.

### Quantitative PCR analysis

Quantitative PCR analysis of three phyla (*Firmicutes*, *Bacteroidetes*, *Actinobacteria*) was performed using previously published primers (Table 1) [38], [39]. PCR reactions were performed in a final volume of 20 µl using Brilliant II Ultra-Fast SYBR Green qPCR Master Mix 2X (Agilent Technologies, Palo Alto, CA), in presence of 10 ng of template DNA, following the manufacturer's instructions. Quantitative PCR reactions were performed on the Mx3005P (Agilent Technologies, Palo Alto, CA). The thermocycling protocol consisted of an initial denaturation step at 95°C for 10 min followed by 40 cycles of denaturation at 95°C for 30 s, annealing at 61°C for 30 s and elongation at 72°C for 30 s, followed by a final step producing a dissociation curve. Data analysis was achieved using the Mx Pro qPCR software (Agilent Technologies, Palo Alto, CA).

**Table 1:** Primers used for qPCR analysis of the samples.

Name	Sequence 5′-3′	Target	Annealing temp. (°C)	Source
BAC338F	ACTCCTACGGGAGGCAG	Total bacteria	61	[39]
BAC516F	GTATTACCGCGGCTGCTG			
789cfbF	CRAACAGGATTAGATACCCT	*Bacteroidetes*	61	[38]
cfb967R	GGTAAGGTTCCTCGCGTAT			
Act920F3	TACGGCCGCAAGGCTA	*Actinobacteria*	61	[38]
Act1200R	TCRTCCCCACCTTCCTCCG			
928F-Firm	TGAAACTYAAAGGAATTGACG	*Firmicutes*	61	[38]
1040FirmR	ACCATGCACCACCTGTC			

### Statistical analyses

Pearson correlation and one-way ANOVA with Kruskal-Wallis test and figures were performed using GraphPad Prism V 5.0 for Windows (GraphPadSoftware, San Diego, CA). Shannon's diversity index and Ward's hierarchical clustering for the samples were obtained using the Paleontological Statistics (PAST) software [40].

## Results

### HuGChip development and probe design

The database used for probe design was initially developed by Rajilić-Stojanović *et al.* [8] and completed to achieve a curated database of 1,052 16S rRNA gene sequences, each corresponding to a distinct phylotype. The PhylArray probe design strategy (Figure 1) was used for each family in order to take into account the sequence polymorphism (available at http://g2im.u-clermont1.fr/HuGChip/). Five non-overlapping 25-mer regions were selected within each family. For each, the number of non-degenerate combinations varied from 1 up to 182, encompassing explorative probes. Such probes should, therefore, allow the detection of undescribed microorganisms belonging to the targeted taxon. This resulted in a set of 4,441 probes (Table S1), spotted in triplicates and targeting 66 families (Table 2). The specificity of each probe was tested against the curated database: 2,442 probes were specific and 1,919 were explorative. The remaining 80 probes were redundant, meaning probes which could cross-hybridize with sequences of different families. Among them, 62 hybridized with sequences from families of the same order of the original target (Table S2). Next, the probe set was also verified using the Greengenes and SILVA databases, leading to respectively 1,852 and 1,486 specific probes. This decrease is likely due to a comparison with an exhaustive repertoire of bacterial sequences, encompassing those from families unexpected or absent in the gut environment. Among the originally defined explorative probes, only 164 and 206 had counterparts in respectively Greengenes and SILVA databases, therefore justifying the word “explorative” for all the remaining probes. The explorative probes which had counterparts in the databases were mostly specific for the intended family (respectively 141 and 136 probes accordingly to Greengenes and SILVA). The remaining 23 or 70 probes were specific for the order (Greengenes, 16 probes; SILVA, 30 probes), the class (none for Greengenes; 9 for SILVA) or the phylum (2 for Greengenes; 10 for SILVA).

**Table 2:** Phyla and families of the human gut microbiota targeted by the HuGChip.

Phylum	Family	Number of probes	Phylum	Family	Number of probes
Actinobacteria	*Actinomycetaceae*	36	Firmicutes	*Lactococcaceae*	44
	*Bifidobacterium*	44		*Leuconostocaceae*	36
	*Coriobacteriaceae*	65		*Staphylococcaceae*	10
	*Corynebacteriaceae*	26		*Streptococcaceae*	98
	*Micrococcaceae*	11		Unclassified Firmicutes	59
	*Propionibacteriaceae*	13		Uncultured clostridiales I-A	95
	**TOTAL**	***195***		Uncultured clostridiales I-B	38
Bacteroidetes	*Bacteroidaceae*	109		Uncultured clostridiales II	69
	*Porphyromonodaceae* A	27		**TOTAL**	***2323***
	*Porphyromonodaceae* B	38	Fusobacteria	*Fusobacteriaceae*	56
	*Porphyromonodaceae* regrouped	94		**TOTAL**	***56***
	*Prevotellaceae*	129	Lentisphaerae	*Victivallaceae*	5
	*Rikenellaceae*	49		**TOTAL**	***5***
	Uncultured Bacteroidales I	43	Proteobacteria	*Aeromonodaceae*	54
	Uncultured Bacteroidales II	19		*Alcaligenaceae*	46
	**TOTAL**	***508***		*Burkholderiaceae*	56
Cyanobacteria	Unclassified A	35		*Campylobacteraceae*	45
	**TOTAL**	***35***		*Desulfovibrionaceae*	21
Firmicutes	*Aerococcaceae*	50		*Enterobacteriaceae*	205
	*Bacillaceae* A	70		*Helicobacteraceae*	16
	*Bacillaceae* B	70		*Moraxellaceae*	35
	*Bacillaceae* regrouped	86		*Neisseriaceae*	117
	*Carnobacteriaceae*	64		*Oxalobacteriaceae*	46
	Clostridium Cluster I	115		*Pasteurellaceae*	93
	Clostridium Cluster III	28		*Pseudomonodaceae*	12
	Clostridium Cluster IV	165		*Succinivibrionaceae*	23
	Clostridium Cluster IX	198		Unclassified B	25
	Clostridium Cluster XI	127		Unclassified Rhizobiales	42
	Clostridium Cluster XIII	75		Unclassified Sphingomonadales	137
	Clostridium Cluster XIV	324		*Vibrionaceae*	102
	Clostridium Cluster XV	30		*Xanthomonodaceae*	116
	Clostridium Cluster XVI	55		**TOTAL**	***1191***
	Clostridium Cluster XVII group 1	7	Spirochaetes	*Brachyspiraceae*	12
	Clostridium Cluster XVII group 2	43		**TOTAL**	***12***
	Clostridium Cluster XVIII	86	Tenericutes	*Anaeroplasmataceae*	59
	*Enterococcaceae*	14		**TOTAL**	***59***
	Incertae Sedis 11	46	Verrucomicrobia	*Verrucomicrobiaceae*	57
	*Lactobacillaceae*	221		**TOTAL**	***57***

### *In silico* explorative probe assessment of the HuGChip

In order to assess the relevance of the explorative probe design strategy, these probes were tested *in silico* with metagenomic data obtained from two human faecal samples. The results indicated that 7 explorative probes could hybridize (100% identity) with metagenomic sequences, 3 with sample 176 and 4 with sample 205. As seen in Table 3, the MG-RAST affiliation of the detected sequences was in agreement with the family the probes targeted. Surprisingly, one MG-RAST affiliation was directly with a referenced strain, therefore not justifying that the probe was effectively explorative (Sequence #176-3): in fact, difference was due to the presence of ambiguous nucleotides (N) in sequences from the microarray database. Furthermore, another sequence (sequence #205-4) was detected *in silico* with a probe targeting the *Streptococcaceae* family while it was affiliated by MG-RAST as an uncultured bacterium (Table 3). When a BLASTN search was performed against the Genbank database, the best hit was with a 16S rRNA gene sequence (accession number: JX079558.1), mentioned as an uncultured *Streptococcaceae*, therefore confirming the effectiveness of this HuGChip explorative probe.

**Table 3:** *In silico* hybridization of HuGChip explorative probes and sequences from two metagenomic samples.

Sample	Sequence ID*	HuGChip Probe	MG-RAST Affiliation
176	176-1	6947_1_10 *Bacteroidaceae*	*Bacteroides uniformis*
	176-2	6947_3_6 *Bacteroidaceae*	*Bacteroides uniformis*
	176-3	7007_1_4 *Verrucomicrobiaceae*	*Akkermansia muciniphila* ATCC BAA-835
205	205-1	6947_3_6 *Bacteroidaceae*	*Bacteroides uniformis*
	205-2	6961_3_7 Clostridium ClusterXVI	*Erysipelotrichaceae bacterium* 5_2_54FAA
	205-3	6965_4_7 *Coriobacteriaceae*	*Collinsella aerofaciens*
	205-4	6989_1_27 *Streptococcaceae*	*Uncultured bacterium*

* The Sequence IDs 176-1 to 176-3 correspond respectively to the metagenomes sequences numbers NODE_13676,NODE_30 and NODE_2236. *The Sequence IDs 205-1 to 205-4 correspond respectively to the metagenomes sequences numbers NODE_141032, NODE_71670, NODE_96151 and NODE_38960.

### Criteria optimization for qualitative and quantitative detection of bacteria

We first decided that a bacterial family would be considered present in a sample if at least 3 of the 5 different 16S-regions showed positive signal as all the 16S rRNA regions are not accessible for hybridization in an homologous manner [41]. Then, to select the best criteria for specific detection, as well for a semi-quantitative determination of bacterial families in samples, the hybridization of a mock community of five known 16S rRNA gene amplicons was performed. This bacterial mix corresponded to 5 different species frequently recovered from gut microbiota, in a defined ratio (Table 4). After hybridization and fluorescent signal acquisition, different signal to noise ratios (SNR) were applied to attribute a positive signal. A SNR equal or superior to 12 gave the result expected (Table 4). Furthermore, when the median of the triplicates was used and an average of the sum of the signals for each of the five regions was calculated, the relative abundance of the bacteria hybridized on the microarray was correlated to the relative abundance in the artificial bacterial mix (Pearson correlation of 0.99). Therefore, hybridization signal superior or equal to 12-fold the level of background noise indicated positive probe hybridization, *i.e.* the presence of at least one 16S-region from a bacterial family. When 3 or more regions for each family were positive with these criteria, the family was claimed present in a relative abundance defined as the mean of the signal obtained for the highest signals for each of the region used to identify the family.

**Table 4:** Relative abundances of bacterial families at different signal to noise ratios (SNR) using a known mix of 16S rRNA amplicons.

			Relative abundances (%)				
		Amount in mix (ng)	SNR≥3	SNR≥5	SNR≥10	SNR≥12	SNR≥15
**Expected Families**	*Bacteroidaceae*	250	18,8	19,0	19,0	22,6	22,6
	Clostridium Cluster IV	350	28,1	28,4	28,4	33,8	33,8
	Clostridium Cluster XIV	200	17,9	18,2	18,2	21,5	21,5
	*Enterobacteriaceae*	100	9,7	9,9	9,9	11,7	11,7
	*Lactobacillaceae*	100	8,6	8,7	8,7	10,4	10,4
	**Total**	1000	83,1	85,2	84,2	100,0	100,0
**Cross-hybridizations**	*Bifidobacterium*		0,2	0,2	0,2		
	Clostridium Cluster IX		12,2	12,3	12,3		
	*Coriobacteriaceae*		3,2	3,3	3,3		
	*Rikenellaceae*		1,3				
	**Total**		16,9	14,8	15,8	0	0

### Comparison of HuGChip and amplicons pyrosequencing data

**Figure 2:**
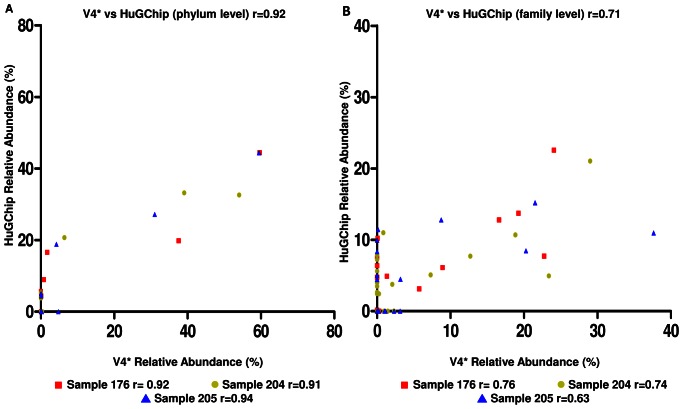
Comparison of relative abundances obtained with pyrosequencing (V4) and the HuGChip at two taxonomic levels. Three samples (▪ 176, • 204 and ▴ 205) were compared at both the phylum and the family level. Pearson correlation coefficients were calculated for each sample. *V4 corresponds to the pyrosequencing of the V4 hypervariable region data.

**Figure 3:**
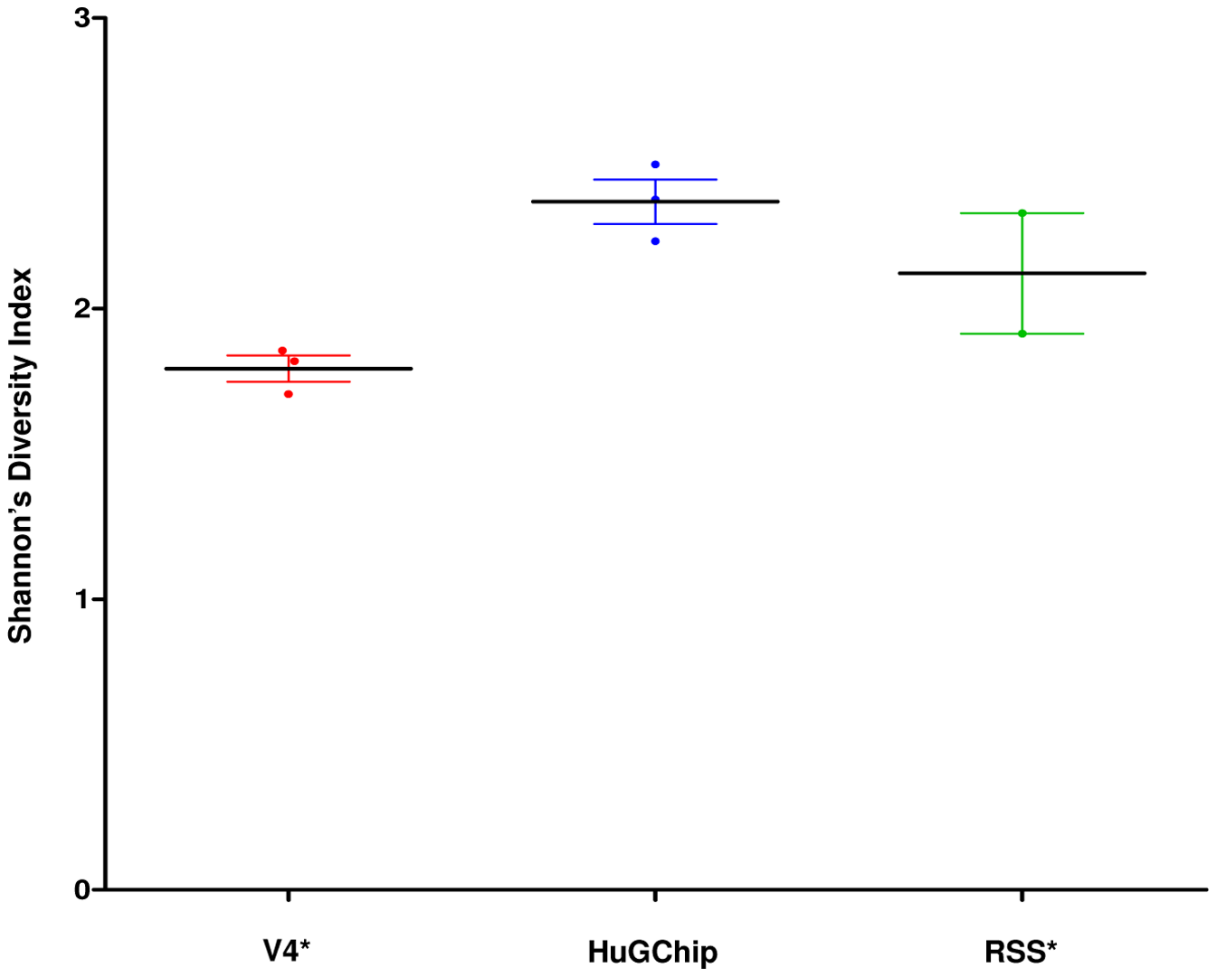
Comparison of Shannon's diversity index derived from the data obtained by pyrosequencing (V4), the HuGChip and metagenomics (RSS) on the faecal samples. *V4 corresponds to the pyrosequencing of the V4 hypervariable region data. **RSS corresponds to the Random Shotgun Sequencing data.

DNA extracted from stool samples of 3 patients was characterized in parallel by amplicons pyrosequencing of the V4 hypervariable region of the 16S rRNA gene and the HuGChip. The results were analyzed at two different taxonomic levels, the family and the phylum level. For each taxon, the ratios of numbers of RDP classified sequence reads were compared with their corresponding relative abundance obtained with the microarray. Hierarchical clustering at family level for both techniques showed exactly the same clustering pattern (Figure S1). Following this result, Pearson's coefficients were calculated as a measurement of linear correlation between sequence-based RDP assignments ratios versus HuGChip relative abundance of all common taxonomic groups for the phylum and family (Figure 2). The results at the phylum level showed a high average Pearson's correlation coefficient (average r = 0.92, ranging from 0.91 to 0.94). At the family level the correlation coefficients still showed a positive correlation with an average Pearson's correlation coefficient of r = 0.71 (ranging from 0.63 to 0.76). The differences resulted from families which were detected by one technique but not the other: the family not detected by the HuGChip represented an average over the 3 samples of 5.6% of the total ratios, whereas the families detected by the HuGChip, but not by pyrosequencing, represented an average of 23.5% of the relative abundances. Another result was the sequences respresenting families labelled “unclassified” (e.g. unclassified Rhizobiales, unclassified Clostridiales I-A…) presented an average relative abundance varying from 18.3% to 30.2% between the HUGChip and the pyrosequencing analysis respectively. Consequently, given these results, Shannon diversity indexes were calculated showing higher indexes with the HuGChip than with pyrosequencing (Figure 3), even if considered as statistically non-significant (one way ANOVA, Kruskal-Wallis test p = 0.062).

### Comparison of the HuGChip with metagenomic data

**Figure 4:**
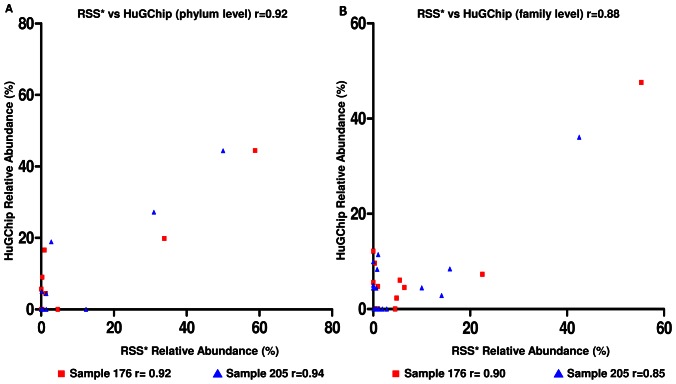
Comparisons of relative abundances obtained with metagenomic (RSS) and the HuGChip at two taxonomic levels. Two samples (▪176 and ▴ 205) were compared at both the phylum and the family level. Pearson correlation coefficients were calculated for each sample. *RSS corresponds to the Random Shotgun Sequencing data.

In order to avoid eventual bias from analyses limited to the V4 region, together with amplification bias, two of the samples mentioned above were also analyzed using random shotgun sequencing with two different levels of coverage: 14,869 sequences were obtained for the samples 176, and ∼10 fold more for the sample 205 (140,766 sequences). This allowed two different sequencing depths in identified 16S rRNA features as provided by MG-RAST: 598 sequences for sample 176 and 1,458 for sample 205. The SILVA database was used to affiliate features at the phylum and family levels and results were compared to the HuGChip hybridization signals using the above criteria. Pearson correlation indicated a high similarity at both phylum and family level between the two technical approaches. As indicated in Figure 4, the average Pearson's correlation coefficient was of 0.93 at the phylum level (respectively of 0.92 and 0.94 for samples 176 and 205) and of 0.88 at the family level (respectively 0.90 and 0.85). The Greengenes and RDP databases were also used to compare the two techniques and revealed similar Pearson's correlation coefficients (data not shown). As previously, the differences relate (i) to the difficulty for the DNA microarray to detect some rare taxa, and (ii) to families detected with a relatively high abundance by the microarray which are not detected in the metagenomes. The abundance results of the three techniques were compared with qPCR for three main phyla present in the gut microbiota.

### Quantitative PCR analysis and comparison with HuGChip

**Figure 5:**
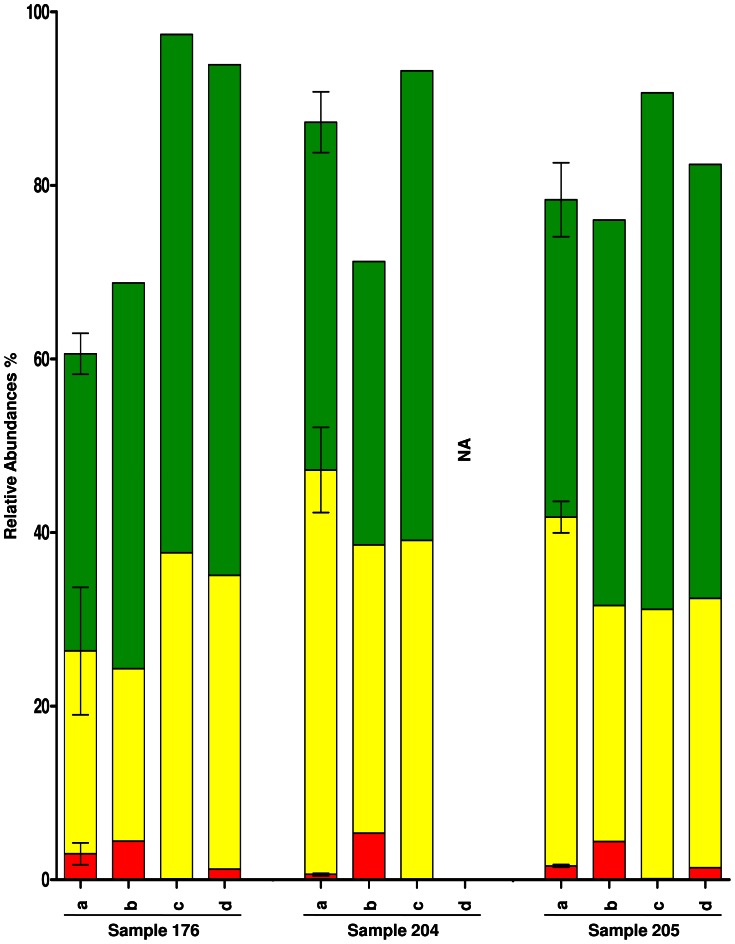
Comparison of qPCR results with results obtained with the HuGChip, pyrosequencing and metagenomics. The phyla *Actinobacteria* (red), *Bacteroidetes* (yellow) and *Firmicutes* (green) were analyzed by (a) qPCR (n = 3), (b) HuGChip, (c) pyrosequencing and (d) metagenomics. *NA corresponds to “not available”.

The qPCR technique was used here as a benchmark for quantitative analysis of the two most dominant phyla (*Firmicutes* and *Bacteroidetes*) present in faecal samples and a less abundant one (*Actinobacteria*). The results obtained confirmed that relative abundances vary slightly between the different techniques. The sequencing of the V4 region showed the highest abundances for the *Firmicutes* phylum and the HuGChip had the lowest relative abundance in only one sample (Figure 5). For the *Bacteroidetes* phylum (Figure 5), the HuGChip showed, for the three samples, the lowest abundances compared to the other techniques. Finally, it can be seen that bacterial species from the phylum *Actinobacteria* seem to be under-estimated as they are not detected with the pyrosequencing technique whereas they are detected with the three other techniques, the HuGChip giving the highest abundance (Figure 5).

## Discussion

A rapid evaluation of the composition of the human gut microbiota is becoming essential in order to gain a better understanding of the interactions with the host, for example in the context of diseases, infections, ageing, or nutrition. In this study, we present a phylogenetic microarray designed at the family level that is able to assess the human gut microbiota composition. Even if differences observed between two samples at this taxonomic rank may be biologically difficult to interpret, due to functional diversity within a family, this tool should provide rapid and cheap information about the ratio of bacterial families shared among humans. This microarray was first validated *in silico*, and then optimal data interpretation regimes were empirically determined using a mock community made from reference bacterial species that inhabit the human gut. These criteria are very important as the signal to noise ratio (SNR) (as well as the number of regions considered positive) influences the qualitative and quantitative analysis of the microarray data (see Figure S2 as an example). The microarray was finally hybridized with 16S amplicons from complex samples and the results were compared with data from three other culture independent techniques applied to the same DNA samples: 454 pyrosequencing of the V4 hypervariable region of the 16S rRNA gene, metagenomic shotgun sequencing and qPCR of three selected phyla.

Microarrays are recognized as fast and user-friendly approaches to study bacterial communities [16]. Several phylogenetic microarrays have been developed to evaluate the presence and relative abundance of known bacteria from the whole human gut microbiota [8], [19], [42]. In contrast to other microarrays, the HuGChip, with its probe design strategy, is a phylogenetic microarray which targets known bacteria, together with potent uncharacterised respresentatives of the corresponding families. Furthermore, the design strategy allows, for each family, the determination of five regions along the 16S rRNA gene, which are not pre-defined as for example in the HITChip strategy, but are selected to give the best reliability on microarray data analysis. Twenty-five mer probes have been shown to give the best specificity [28], [43] and thus, were selected for the HuGChip. Their specificities were first verified *in silico* using the sequence database used for the design indicating that the large majority of probes could be classified as specific or explorative. A small number of redundant probes were detected. These probes were frequently specific of the taxonomic levels above the family (e.g. class or order). Such hierarchical hybridization has been reported previously for other microarrays [21]. Furthermore, the probes were compared against bacterial databases containing sequences from different environments (e.g. soil, water, air, and human microbiota). The consequence was a decrease in probe specificity that might be attributed to bacterial species which had not been described in the human gut microbiota. Most of the explorative probes would not target known species, some (64 and 206 respectively for Greengenes and SILVA) could target known bacteria which were not originally detected in the human gut microbiota. Consequently, accordingly to Greengenes, only 23 of the total explorative probes were identified as hybridizing sequences from bacterial representatives from another family, including 5 targeting another phylum. These numbers rose to respectively 70 and 21 representatives using SILVA data. In fact, these results are likely over-estimates as the human gut does not host all the bacterial identified so far in all the environments. Moreover, this could explain the different relative abundance of the unclassified sequences between the pyrosequencing of the V4 hypervariable region of the 16S rRNA gene sequence and the HuGChip (respectively 30,2% and 18,3%). Moreover, it was shown that 7 of the explorative probes of the HuGChip harboured 100% sequence identity and a correct taxonomic affiliation at the family level with sequences from the two metagenomes justifying their presence and benefits. These results showed that the probe design helped in minimizing the main limitations of microarrays: the detection of species which were not yet described and/or which were not included in databases used for the probe design. Other microarrays limitations could be caused by the presence of ambiguous nucleotides (N) in sequences from databases due to sequencing bias and errors: these were also at least partly overcome in this study with the use of the HuGChip explorative approach. Using this strategy, the cross-hybridization of a sequence from another family cannot be excluded but is rather unlikely and if sometimes real, contributes weakly to the overall signal, at least an order of magnitude less [20].

Using a mock community composed of 5 different families allowed setting the best threshold which had to be used with the HuGChip to analyze gut microbiota samples. As it has been shown that there are strong variations of hybridization signal intensity from probe-target duplexes with similar predicted duplexes [21], [35], [40], [44], [45], at least three of the five regions for each family have to show a probe signal to noise ratio above 12 to be considered present in the sample. These defined parameters helped to reduce the impact of possible cross-hybridizations and showed the best specificity and sensibility.

Next generation sequencing through amplicon-based or random shotgun sequencing as well as qPCR are other culture-independent techniques used to study complex ecosystems. To further evaluate the application of the HuGChip, human faecal samples were analyzed and results were compared to these culture-independent techniques on the same samples.

Pyrosequencing of amplicons from variable regions of the 16S rRNA gene provides a deep, fast, quantitative analysis and allows the identification of unknown bacteria [4], [46]–[49]. Although this technique specifically focuses on a hypervariable region of the 16S rRNA gene, whereas the HuGChip targets 5 regions for each family, these different approaches generated similar profiles at both the phylum and family levels. This has been already observed between the pyrosequencing of the V4 and V6 hypervariable region amplicons and the HITChip [22]. More recently, the pyrosequencing of the V1 to V6 hypervariable region amplicons of faecal and ileum lumen-content was compared with results obtained with the HITChip [23] and similar coefficients were also obtained.

Although the profiles were similar, relative abundance results between the techniques vary; it was likely due to the different means used to quantify each family, one based on sequence hit, the other on probe signal and each having their own bias [50]–[52]. While possible cross-hybridization or sequencing errors affect bacterial detection, incorrect or obsolete classification, annotation of sequences can also induce discrepancies. In our study, pyrosequencing of the V4 region of the 16S rRNA gene provided an important amount of unclassified sequences, part of which may have been detected and affiliated to a family due to the presence of explorative probes on the microarray. Previous studies have already shown that microarrays detected bacterial genus that were ignored by pyrosequencing of the V1 to V6 16S hypervariable regions of the 16S rRNA gene [23]. Moreover, the use of different primer sets for the HuGChip experiments and the pyrosequencing of the V4 hypervariable region may also likely contribute partly to the discrepancy observed in these two methods.

Random shotgun sequencing referred as metagenomics is another alternative culture-independent technique to study the gut microbiota, whose main advantage is the determination of large amounts of sequences from total DNA, in a more direct way, thereby avoiding PCR bias. As it does not target a particular single gene, this technique has proven to be very powerful, helping with the study of the ecosystems' metabolic potentialities and diversity [53]–[57]. To the best of our knowledge, this is the first time microarray data was compared to metagenomics in the perspective to address the diversity of the samples. Once again, high correlations were obtained at both phylum and family levels when the 16S gene sequences from the metagenomes were analyzed. These correlations were equivalent or even higher than the coefficients obtained between pyrosequencing and the microarray. The minor differences observed between the two techniques were certainly attributed to 2 congruent reasons: the microarray's sample preparation procedure (necessitating PCR, and consequently a potent quantitative bias) and the low number of ribosomal sequences available for taxonomic attributions from the metagenomic results (around 1,500 for the deepest sequenced sample).

The results of the three techniques were finally compared to qPCR at the phylum level. This is a commonly used technique to quantify specific taxonomic groups in a sample. Even if differences were seen among the techniques for the three phyla tested, they were likely due to the low number of experiments and that all the techniques present globally similar abundance patterns. The microarray gave a higher signal for the low-represented phylum (*Actinobacteria*) compared to 16S pyrosequencing and metagenomics, near to qPCR values. Taken into account that primers used in this study to amplify 16S rRNA gene sequences of the samples should rather lead to an underestimation of *Bifidobacterium spp* from the phylum *Actinobacteria*, it remains to be determined whether this is due to this particular taxonomic group or to the fact that it corresponds to a low-represented phylum, which is under-detected with pyrosequencing methods. Taken into account that the HuGChip gave higher Shannon Diversity Index when compared with either 16S pyrosequencing or metagenomics argues preferentially for a better evaluation of low-represented families while dominant ones (*Firmicutes* and *Bacteroidetes*) seemed to be less prevailing.

Altogether, the results showed that the HuGChip is a suitable tool to assess the human gut microbiota. Contrary to other microarrays, this tool contains explorative probes which allow the detection of unknown bacteria, without providing strong taxonomic evidences, but probably contributes to a better detection of low-represented families, and increases the specificity at the family level thanks to the use of 5 different regions per family. Pyrosequencing of the V4 region of the 16S rRNA gene provided an important amount of unclassified sequences, part of which may have been detected and affiliated by the microarray to a family: in fact, the presence of explorative probes based on 5 specific “regions” spread along the 16S rRNA gene and not restricted to a small variable region is a significant improvement as a majority of the explorative probes do not show counterparts in international database used for the affiliation of sequencing data. This suggests also that the microarray could be used for other environments, in which bacterial families are similar: this encompasses samples from other compartments of the digestive tract that have different bacterial compositions [49] and that partially explain the discrepancies between the HITChip and pyrosequencing of the V1 to V6 16S hypervariable regions observed in a previous study [23]. This might be avoided by using the HuGChip, which could evaluate the microbiota from these different compartments in the human host, but also in other animals (e.g. rodents, ruminants).

In this study, we showed that the HuGChip had similar profiles at both the phylum and the family level. This microarray can thus be considered as a suitable tool to analyze the human gut microbiota as it is a rapid, cheap and user friendly technique which allows studying several samples in parallel. Currently, the format and design of the HuGChip (8×15k probes, three probe replicates) make it possible to analyze 16 different samples per run reducing costs and limiting inter microarray bias. Furthermore, the analysis of the data extracted from the microarray is not laborious compared to other high throughput techniques and stands on 5 different regions per family, increasing specificity. Microarrays are also a particularly well-adapted format to monitor the gut bacterial environment over the time and are a mean to give an alternative determination of the bacterial richness and abundance of a sample. Taken altogether, this suggests that the microarray should also be used to characterize and select the samples of interests in order to study them with next generation sequencing techniques. Especially, improved techniques such as MiSeq Illumina technology or emerging third generation sequencing which may bring increased depth of analysis with lower time of analysis, and will surely provide new knowledge of the gut microbiota's composition, structure and role within the human health.

## Supporting Information

Figure S1 **Impact of threshold selection on the results of a complex sample.**
(PPTX)

Figure S2 **Euclidean clustering of the three samples when they are analyzed by (a) pyrosequencing and (b) the HuGChip.**
(PPTX)

Table S1 Targeted family, sequence, region and localization on the 16S rRNA gene of the probes spotted on the HuGChip.
(XLSX)

Table S2 Greengene Database (July 2011).
(XLSX)
