# Supplementary material for: The Human Gut Chip “HuGChip”, an Explorative Phylogenetic Microarray for Determining Gut Microbiome Diversity at Family Level
Source: PLoS One. 2013 May 17;8(5):e62544. doi: 10.1371/journal.pone.0062544 (PMC3656878; doi:10.1371/journal.pone.0062544)
Supplement: Figure S1 — Impact of threshold selection on the results of a complex sample. (PPTX) [file pone.0062544.s001.pptx]

## Slide 1
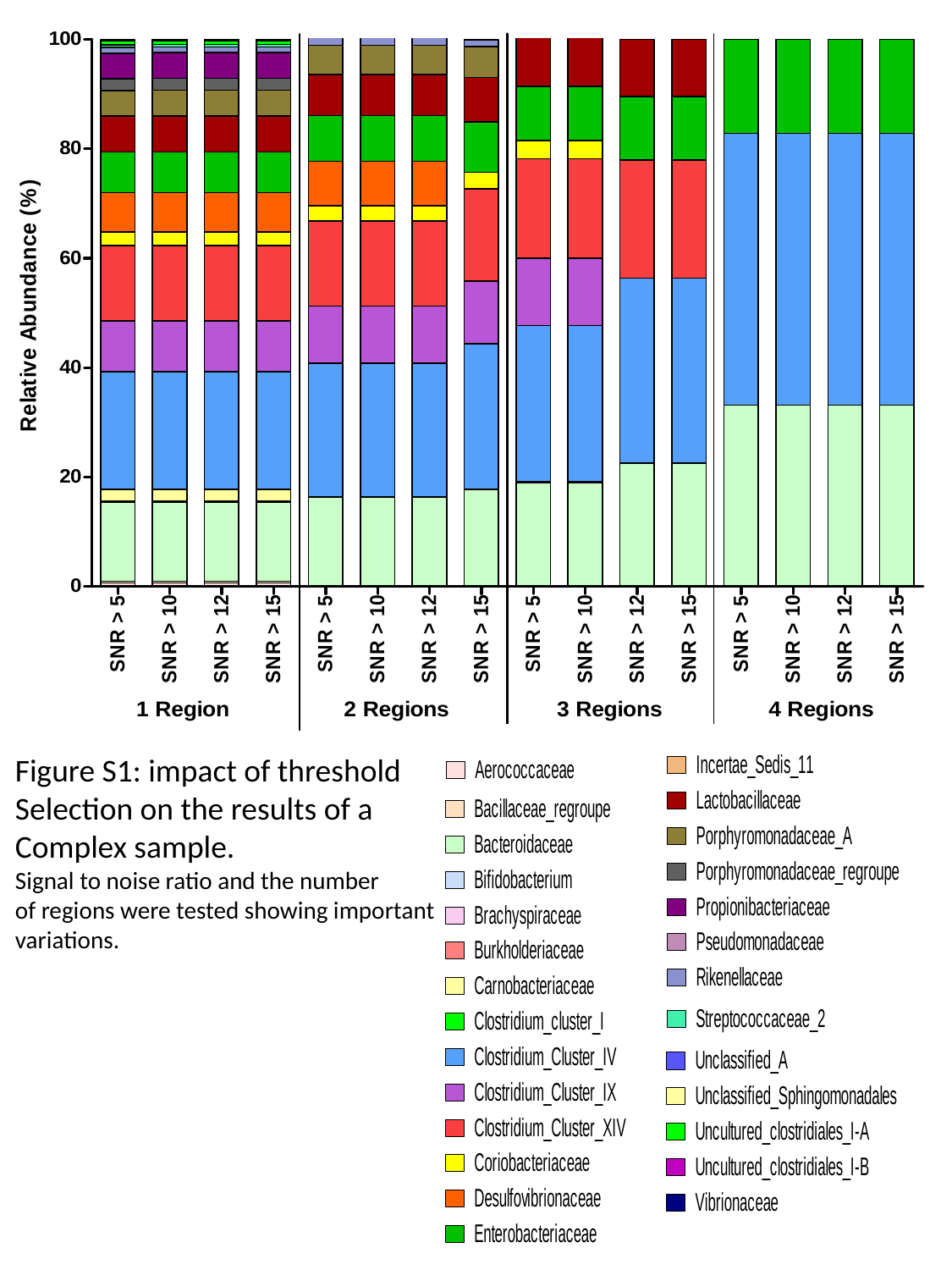

Figure S1: impact of threshold
Selection on the results of a
Complex sample.
Signal to noise ratio and the number
of regions were tested showing important
variations.
