# Supplementary material for: The Human Gut Chip “HuGChip”, an Explorative Phylogenetic Microarray for Determining Gut Microbiome Diversity at Family Level
Source: PLoS One. 2013 May 17;8(5):e62544. doi: 10.1371/journal.pone.0062544 (PMC3656878; doi:10.1371/journal.pone.0062544)
Supplement: Figure S2 — Euclidean clustering of the three samples when they are analyzed by (a) pyrosequencing and (b) the HuGChip. (PPTX) [file pone.0062544.s002.pptx]

## Slide 1
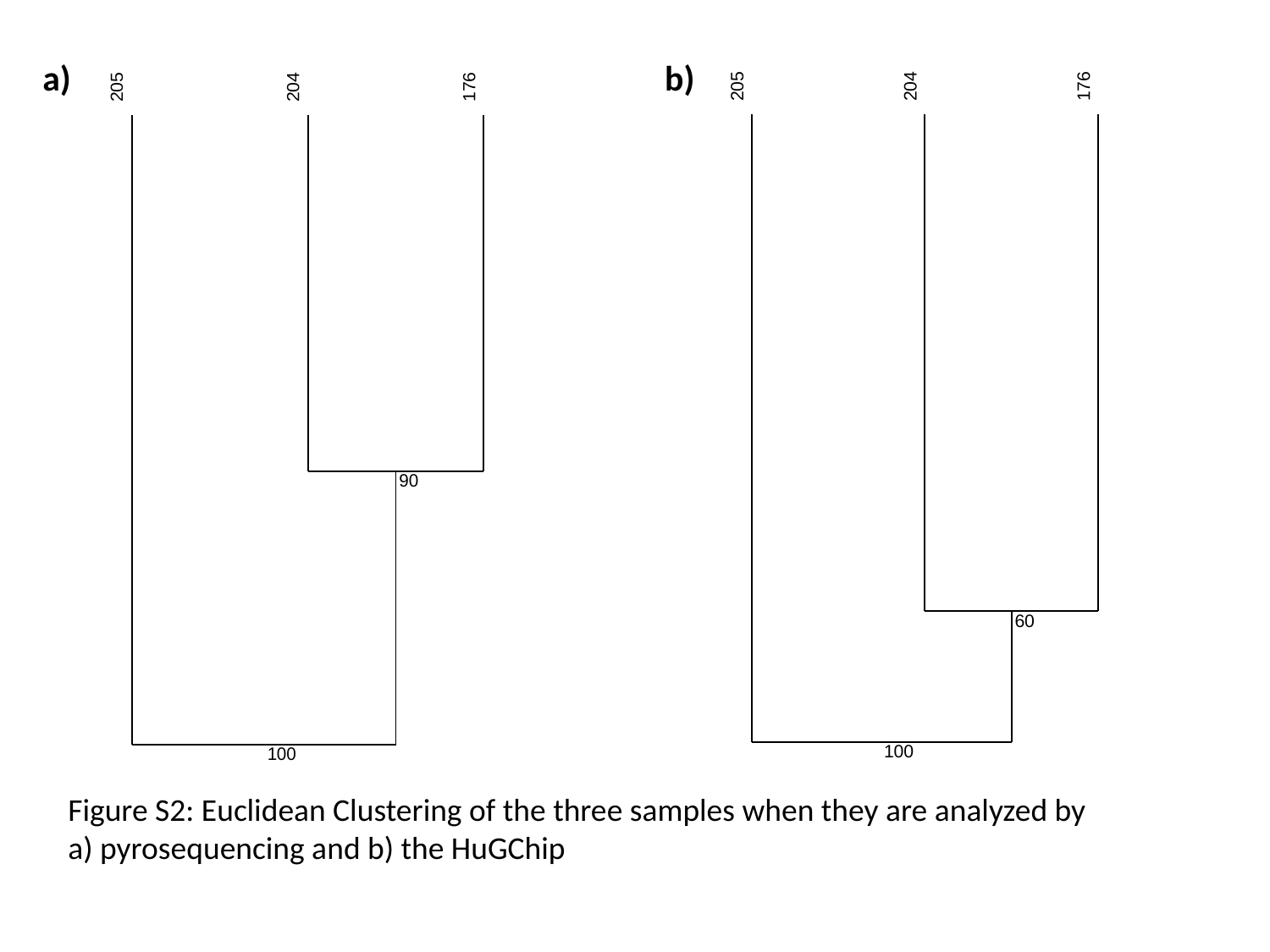

a)
b)
Figure S2: Euclidean Clustering of the three samples when they are analyzed by
a) pyrosequencing and b) the HuGChip
